# An Overview of Angiogenesis in Bladder Cancer

**DOI:** 10.1007/s11912-023-01421-5

**Published:** 2023-04-13

**Authors:** Ghada Elayat, Ivan Punev, Abdel Selim

**Affiliations:** 1 Department of Natural Science, Middlesex University, London, UK; 2 Department of Histopathology, Tanta University, Tanta, Egypt; 3 Histopathology Department, King’s Health Partners, King’s College Hospital, London, UK

**Keywords:** Bladder cancer, Angiogenesis, Microenvironment, Vascular mimicry, Non-coding RNAs, VEGF

## Abstract

**Purpose of the Review:**

Angiogenesis plays a key role in bladder cancer (BC) pathogenesis. In the last two decades, an increasing number of publications depicting a multitude of novel angiogenic molecules and pathways have emerged. The growing complexity necessitates an evaluation of the breadth of current knowledge to highlight key findings and guide future research.

**Recent Findings:**

Angiogenesis is a dynamic biologic process that is inherently difficult to assess. Clinical assessment of angiogenesis in BCs is advancing with the integration of image analysis systems and dynamic contrast-enhanced and magnetic resonance imaging (DCE-MRI). Tumour-associated macrophages (TAMs) significantly influence the angiogenic process, and further research is needed to assess their potential as therapeutic targets. A rapidly growing list of non-coding RNAs affect angiogenesis in BCs, partly through modulation of vascular endothelial growth factor (VEGF) activity. Vascular mimicry (VM) has been repeatedly associated with increased tumour aggressiveness in BCs. Standardised assays are needed for appropriate identification and quantification of VM channels.

**Summary:**

This article demonstrates the dynamic and complex nature of the angiogenic process and asserts the need for further studies to deepen our understanding.

## Introduction

Bladder cancer is the tenth most common malignancy globally and the sixth most common cancer in men [1]. Most BCs originate from the urothelium, and the most common type is known as urothelial carcinoma or transitional cell carcinoma. Less common types include adenocarcinoma and squamous cell carcinoma [2].

Approximately, 70% of urothelial carcinomas present as superficial cancers confined to the mucosa with a high recurrence rate following transurethral resection. About a third of non-muscle invasive cancers will progress to higher grade more invasive tumours. Muscle-invasive carcinomas are usually associated with metastatic disease and carry a poor prognosis [3]

Angiogenesis is a key step in tumour survival and progression in BCs. Vascular endothelial growth factor (VEGF) has been widely investigated due to its vital role in BC angiogenesis, and its expression has been mostly associated with worse outcomes [3, 4]. Studies investigating angiogenesis in BCs are expanding, and current knowledge projects an increasingly complex picture supporting the need for better understanding of the biologic concepts of angiogenesis to improve disease characterisation and patient-targeted therapeutic strategies.

In this article, we discuss the methods employed in the clinical assessment of angiogenesis in BCs and highlight existing limitations. Of particular interest, we highlight the various angiogenic molecules that have emerged in the past two decades. We discuss the role of the tumour microenvironment and the impact of inflammation and TAMs on the angiogenic process. We shed light on alternate regulatory mechanisms such as non-coding RNAs. We finally bring to attention unconventional forms of angiogenesis as we discuss VM and its significance in BCs.

## Clinical Assessment of Angiogenesis in Bladder Cancers

Angiogenic activity in BCs has been long employed as a parameter for the characterisation and evaluation of tumour behaviour and aggressiveness. Assessment of angiogenesis is understandably a complex process owing to its innate dynamic nature. Micro-vessel density (MVD), as the end product of the angiogenic process, has been the most used method to assess angiogenic activity in BCs. Various angiogenic molecules as drivers of the angiogenic process have been evaluated for potential use as predictors of prognosis. Alternatively, imaging techniques have been shown to be the most valuable tools in assessing vascularity in clinical trials offering both serial and spatial assessment of the process.

### Micro-Vessel Density

MVD has long been used as a prognostic parameter in many cancers. In 1991, Weidner et al. introduced the idea of MVD for the assessment of tumour angiogenesis. MVD describes the number of blood vessels within a defined number of microscopic fields “hotspots” [5]. In BCs, MVD has been correlated with various clinical aspects such as increased mortality and recurrence [6–8]. MVD has also been associated with the expression of various biological markers including p53, VEGF-C, TSP-1, AT1R, Maspin, nitric oxide, and DNA ploidy, to name a few [4, 9–11].

The hotspot technique is mostly used to quantify intra-tumoural blood vessels identified by pan-endothelial cell markers such as CD34, CD31, or von Willebrand factor (vWF). Hotspots are identified by light microscopy, and individual micro-vessels are counted at high magnification in the identified spots [6, 7]. A systematic review in 2014 has shown that high MVD was significantly associated with poor survival [8]. Despite general agreement across the literature, the technique has not yet reached the level of evidence for widespread routine clinical utilisation. Inter-observer variability of vessel counting remains a significant barrier requiring the application of strict counting rules and objective training of individual observers. The diverse nature of studied samples (transurethral resections vs cystectomy specimens) and the likelihood of unrepresentative sampling of micro-vessel hotspots in fragile papillary tumours also contribute to the limited utilisation of MVD as a prognostic parameter [12••].

Intra-tumoural MVD represents the collective interplay between pre-existing vasculature and neovascularisation. Double immunostaining with endothelial cell-specific antibodies such as CD34 or Factor VIII and cycling nuclei-specific antibodies such as Ki67 or proliferating cell nuclear antigen (PCNA) had been employed to highlight ongoing angiogenesis [13].

Novel markers offering the ability to quantitatively distinguish between tumour neovascularisation and pre-existing vessels have been identified. Notably, endoglin (CD105), an accessory receptor for transforming growth factor beta (TGF-β), has been shown to be overexpressed in vascular endothelial cells of tissues undergoing active angiogenesis such as regenerating and inflamed tissues or tumours [14]. Furthermore, levels of endoglin/CD105 have been shown to positively correlate with the extent of endothelial cell proliferation and with the expression of proliferation markers in tumour endothelia [15–17]. Intra-tumour micro-vessel density assessed by endoglin/CD105 staining has been shown to strongly correlate with prognosis in many cancer patients [15–19].

Miyata et al. compared the performance of CD31, CD34, and CD105 in a series of urothelial cancers. CD105 MVD showed the strongest association with stage, grade, and VEGF-A expression [20]. Agrawal et al. demonstrated an association between VEGF, CD105, and p21 expression in 90 cases of non-muscle invasive bladder cancers (NMIBCs) where combination profiles of the three markers significantly predicted hazard for recurrence [21]. A final study investigated the expression of CD105 in 50 biopsies of urinary bladder carcinomas and 15 benign bladder biopsies from Iraqi patients and concluded that CD105 expression was significantly associated with grade in urinary bladder carcinomas [22].

A recent study projects endoglin as a crucial molecule in the determination of an immunosuppressive tumour microenvironment, mainly because of its role on angiogenesis but also in inflammation and in cancer-associated fibroblast (CAFs) biology. Endoglin expression in patient biopsies could be an excellent biomarker of the immunosuppressive tumour microenvironment and may predict patients’ response to immunotherapy [23•]. Considering the limited number of published studies, the potential of endoglin as a therapeutic target and prognostic marker remains to be under-investigated in BCs.

MVD counts represent the combined product of all angiogenic pathways and as such are useful indicators of the effectiveness of anti-angiogenic therapy. Automation of counting methods with the use of image analysis systems promises increased speed and reliability. The development of algorithms for quantification of blood vessels supported by a user-friendly graphical interface can facilitate histopathological assessment and increase reproducibility [24••]. The requirement of obtaining serial biopsy samples during treatment is, however, invasive and subject to inaccuracy due to regional heterogeneity within individual tumours [25].

### Key (Conventional) Angiogenic Molecules

Evolving neovascularisation in tumours is the result of complex dynamic processes involving an imbalance between pro- and anti-angiogenic factors. The main signalling molecules of vascular morphogenesis include VEGF, angiopoietins (ANGPTs), ephrins, TGF-β, and platelet-derived growth factor (PDGF). The anti-angiogenic factors include angiostatin, endostatin, interferon, platelet factor 4, thrombospondin, prolactin 16 kD fragment, and tissue inhibitors of metalloproteinase-1, 2, and 3 [26].

Among the many studied angiogenic factors, VEGF plays a significant and principal role in BC angiogenesis. Elevated levels of VEGF expression are generally associated with worse outcomes for patients with BC [4, 27–31, 32•]. Notably, the level of VEGF expression did not predict outcomes for patients with BC in many studies [33–37]. A meta-analysis including 11 studies showed that elevated levels of VEGF are associated with poor prognosis in patients with BC [37]. The authors, however, highlight several important limitations in interpreting their studies of which heterogeneity emerged as a key factor. Immunohistochemistry, ELISA (enzyme-linked immunosorbent assay), Western blot, and RT-PCR are among the commonly employed detection techniques. Various anti-VEGF antibodies (VEGF A, C, and D) are used. Variability of studied samples is also noted (urine, serum, and tissue samples). Collectively, these factors contribute to the difficult interpretation of the studies [37].

Other markers including basic fibroblast growth factor (bFGF), thrombospondin 1, HIF 1, 2, and 3, interleukin–8 (IL-8), cyclooxygenase-2 (COX-2), and matrix metalloproteinases (MMPs) have also showed promising results in studies correlating these markers with various clinicopathologic parameters [38–40]. A systematic review in 2017 examining the prognostic role of MMPs in BCs indicated that high expression of MMPs was significantly associated with poor prognosis [41]. The authors advice cautious interpretation of the results owing to limitations of the number of included studies reporting on survival parameters, the variability of assays among the studies as well as the use of median/mean values as cut-off point.

### Imaging of Angiogenesis in Bladder Cancers

Several imaging techniques have been developed to analyse tumour vasculature including computerised tomography (CT), positron emission tomography (PET), and ultrasonography. These techniques offer the advantage of serially monitoring spatially localised changes in tumour microcirculations [25]. Dynamic contrast-enhanced and magnetic resonance imaging, dual-energy CT (DECT), and contrast-enhanced ultrasound (CEUS) are among the techniques employed in investigating patients with BC. Of these, MRI is commonly employed in clinical trials of genitourinary tumours [25].

Precise identification of regional angiogenic activity in tumours is a potentially useful tool for guiding treatment selection and monitoring response. Angiogenic imaging poses a significant potential in improving diagnosis, staging, and response monitoring in bladder tumours as evidenced in many clinical trials [25]. DCE-MRI has been shown to be a useful technique able to distinguish leaky, disorganised tumour neo-vessels from mature well-organised vasculature. Studies have demonstrated a positive correlation between DCI-MRI parameters and MVD, histological grade, and stage [25, 42, 43]. The role of angiogenesis imaging in monitoring BC response to treatment has been evaluated in some studies. A study reported that DCE-MRI was significantly more accurate than conventional MRI in predicting a lack of response following 4 cycles of chemotherapy [44]. Another study suggests that DCE-MRI might be a reliable tool in excluding the presence of persistent or recurrent tumours up to 12 months after radiotherapy [45].

Superb microvascular imaging (SMI), an emerging Doppler ultrasound technique, employs an algorithm that has been shown to effectively assess micro-vessels and their distribution. The technique provides valuable information for diseases associated with angiogenesis than other non-invasive techniques. A few studies have demonstrated the effectiveness of SMI in assessing thyroid nodules, breast tumours, and lymph node diseases [46•]. A case report has shown that SMI helped in the detection of a bladder neoplasm avoiding additional cross-sectional imaging [47]. Further studies are needed to assess the role of SMI in monitoring treatment response in BC patients.

## Alternate Factors Influencing Angiogenesis in Bladder Cancers

Decades of research into angiogenic molecules in BCs have unravelled a multitude of potential angiogenic factors and molecules. Functional relation to angiogenesis is demonstrated through association with various classic angiogenic parameters such as MVD and VEGF expression in many of the studies (Table 1). The studies demonstrate the complexity of the regulatory pathways influencing the angiogenic process. Further research is needed to characterise the clinical usefulness and biological significance of individual markers.

**Table 1:** Potential angiogenic markers in BC

Publication	Marker	Samples type	Effect on angiogenesis
Friedrich et al. [9]	Maspin	Tumour samples of patients undergoing transurethral resection (TUR) for bladder carcinoma	Maspin expression is associated with slightly higher vascularisation (no statistical significance)
Oliveira-Ferrer et al. [48]	CEACAM1	BC cell lines RT4, 486p BC tissues samples, severe cystitis, and normal bladder	Downregulation of CEACAM1 in urothelium promotes expression of VEGF-C and -D thus promoting angiogenesis in BC
Patel et al. [49]	DLL4	BC tissue samples of TUR or radical cystectomy and normal tissue samples	DLL4 expression is associated with vascular differentiation in BC
Golshani et al. [50]	HAS-1	Transfected HT1376	HAS-1 interacts with CD44 to promote angiogenesis
Miyake et al. [51]	HO-1	BC specimens T24 BC cell line Xenografts	Inhibition of HO-1 decreases tumour growth and MVD by suppressing angiogenic factors, VEGF and HIF-1α. High expression of HO-1 correlates with high expression of HIF-1α and high MVD but not with VEGF expression
Shirotake et al. [52]	AT1R	TUR tissue specimens	AT1R and MVD are associated with angiogenesis in MIBC
Xue, Lu, and Sun [53]	CD147	BC tissue samples BC cell lines T24, SCaBER, 5637, and normal human urothelial cell line SV–HUC–1	Downregulation of CD147 decreases secretion of MMP-2 and MMP-9 and expression of VEGF
Feng et al. [54]	BLCA-4	BC tissue samples	BLCA-4 may not affect pro-angiogenic pathways in BC, it can however interact with IL-1α, IL-8, VEGF, and MMP-9 to enhance tumourigenesis and tumour invasiveness
Shimada et al. [55]	ALKBH3	BC cell lines UMUC2 and UMUC3 BC tissue samples from TUR or radical cystectomy	ALKBH3 contributes to the development of urothelial carcinomas by accelerating survival, angiogenesis, and invasion through NOX-2-ROS and Tweak/Fn14-VEGF signals
Roudnicky et al. [56]	Endocan	BC tissue samples and normal bladder tissue as well as patient plasma samples HUVEC cell line Transgenic mice	VEGF-A induces endocan expression in vitro and in vivo via VEGF receptor-2 (VEGFR-2). Endocan knockdown in BCs inhibited VEGF-A–induced tube formation, migration, and VEGFR-2 phosphorylation
Beckham et al. [57]	EDIL-3	Human BC cell lines: 5637, TCC-SUP, and T24 Urine samples	Exosomes derived from the urine of patients with BC contain EDIL-3 which drives angiogenesis and cell migration of BC and endothelial cells
Bertz et al. [58]	FGFR3	BC tissue samples	Increased angiogenesis and FGFR protein expression indicate a favourable prognosis in BC. FGFR3 may be able to induce a pro-angiogenic phenotype in urothelial carcinomas
Feng et al. [59]	BLCA1	BC tissue samples	BLCA1 expression correlates with expression of VEGF, MMP9, IL1α, IL8, and MVD, but not with TNFα expression
Gao et al. [60]	KLF5	Cell lines HUVEC, SV40, SVHUC, RT4, WH, BV, 5637, T24, and UMUC3	KLF5 promotes transcription of VEGFA. Inhibition of KLF5 leads to decrease of ERK1/2 and VEGFA
Tian et al. [61]	BAI-1	BC and normal bladder mucosa tissue samples	BAI1 negatively correlates with VEGF, mutant p53, and with MVD
Geng et al. [62]	REG1A	BC tissue samples Xenograft tumours	REG1α expression significantly reduces the proliferation, migration, invasion, and VEGF-induced angiogenesis in vitro
Roudnicky et al. [63]	INSR	BC and normal tissue samples Tumour mouse model	INSR is highly expressed on isolated endothelial cells of invasive BCs and is significantly associated with shorter progression-free and overall survival A positive correlation between GLUT1 and INSR indicates that hypoxia drives INSR expression in tumour-associated blood vessels
Hou et al. [64]	LPXN	BC cell lines BC tissue samples	Overexpression of LPXN significantly promotes the proliferation, invasion, and angiogenesis of BC cells. The impact on tumour progression was abolished by inhibiting PI3K/ AKT signalling pathway
Hui et al. [65]	RASAL2	BC cell lines 5637, 253 J-BV, HUVEC Mice xenografts BC tissue samples	RSAL2 inhibits phosphorylation of AKT thus suppressing ETS1 and VEGF which results in angiogenesis downregulation
Guo et al. [66]	CNF1	BC cell lines Xenograft	CNF1 induces BC cells to secrete VEGF through activating Ras homolog family member C (RhoC), leading to subsequent angiogenesis
Wang et al. [67]	ECM1, FN1, FGF1, FAP, JAM3, THBS1, MFGE8 and COL8A2	RNA-seq data and clinical records of patients with BLCA were collected from the TCGA database. The panel, including 145 genes involved in angiogenesis, was retrieved from the Uniport database and the published work	Single gene expressions of ECM1, FN1, FGF1, FAP, JAM3, THBS1, MFGE8, and COL8A2 involved in angiogenesis and associated with prognosis
Dong et al. [68]	NRP1	The human bladder immortalised epithelium cell line SVHUC1 and BC cell lines including T24, 5637, J82, UMUC3, and RT4	High expression of NRP1 was observed in BC tissues and cells. *NRP1* knockdown promotes apoptosis and suppresses proliferation, angiogenesis, migration, and invasion of BC cells
Gao et al. [69]	YB-1	Radical cystectomy BC tissue samples Human BC cell lines EJ, UMUC3, SW780, RT4, and the human endothelium cell line EA.hy926	YB-1 promotes angiogenesis in BC. High expression of YB-1 is associated with a higher expression of VEGFA
Li et al. [70]	CTSB	Blood samples and tissue samples from BC patients	CTSB is upregulated in tumour tissues and serum extracellular vesicles. Increased exogenous CTSB in endothelial cells by directly ingesting EV-CTSB prominently activates the TPX2-mediated phosphorylation of the AURKA-PI3K-AKT axis, increases VEGFA expression, and promotes angiogenesis
Li et al. [71]	PSMA and CD248	BC specimens from patients who underwent surgery (transurethral resection of bladder tumour (TRUBT) or radical cystectomy Bioinformatics analysis: Data were downloaded from the TCGA portal (https://portal.gdc.cancer.gov/)	PSMA and CD248 are expressed in tumour-associated vessels. Vascular PSMA and CD248 expression levels are significantly associated with several deteriorated clinicopathological features PSMA and CD248 might contribute to angiogenesis and promote further progression of BC
Ma et al. [72]	HSP47	BC tissue samples Bioinformatics analysis using TCGA portal (https://portal.gdc.cancer.gov/)	HSP47 is abnormally overexpressed in BC and is correlated with poor prognosis. HSP47 downregulation suppresses angiogenesis in BC cells. Activation of the ERK pathway and induction of C–C Motif Chemokine Ligand 2 (CCL2) are responsible for HSP47-induced angiogenesis
Wang et al. [73]	Mettl3	BC cell lines T24 and UMUC-3 Transgenic mice	Ablation of Mettl3 in bladder urothelial cells attenuates the oncogenesis and tumour angiogenesis of BC
Abd El-Azeem, Ali and El-Shorbagy [74]	GLUT4	BC tissue samples of radical cystectomies and TUR	GLUT4 and fibroblast activation protein (FAP) expression is significantly associated with increased intratumour MVD and adverse clinicopathological factors
Mori et al. [75]	VCAM-1	Preoperative plasma samples	Elevated VCAM-1 is associated with aggressive BC. Preoperative VCAM-1 may serve as a biomarker to help identify patients likely to benefit from multimodal therapy
Vlachostergios et al. [76]	HIF-2	Publicly available next-generation sequencing (NGS) data from muscle-invasive BC cell lines and patient tumour samples from the MSK/TCGA 2020 cohort were interrogated	HIF-2-altered BC has an aggressive clinical and a distinct genomic and immunogenomic profile enriched in angiogenesis- and immune evasion-promoting genes
Xia et al. [77]	PKM2	Genetically engineered mice BC cell lines RT112, 1376, UMUC3, and T24 and mouse BC cell line MBT2	PKM2 ablation in mouse urothelial cells and/or chemical inhibition of PKM2 reduces complex formation of PKM2 with STAT3, their nuclear translocation, and HIF1α- and VEGF-related angiogenesis
Yang et al. [78]	Occludin	Cell lines T24 and 5637. Human umbilical vein endothelial cells (EA.hy926) and 293 T BC tissue samples	Occludin participates in the development of angiogenesis in BCs by activating the IL8/STAT3 pathway via STAT4

*CEACAM1*, carcinoembryonic antigen-related cell adhesion molecule-1; *DLL4*, delta-like 4; *HAS-1*, hyaluronic acid synthase-1; *HO-1*, haem oxygenase-1; *AT1R*, angiotensin II type 1 receptor; *BCLA*, bladder cancer–specific nuclear matrix protein; *VEGFR*, VEGF receptor; *EDIL-3*, EGF like repeats and discoidin i like domains protein 3; *FGFGR*, fibroblast growth factor receptor; *KLF5*, human Kruppel-like factor 5; *BAI-,1* brain-specific angiogenesis inhibitor-1; *INSR*, insulin receptor; *LPXN*, leupaxin; *RASAL2*, RAS protein activator like 2; *CNF1*, cytotoxic necrotizing factor;1*YB-1*, Y-box binding protein; *CTSB*, cathepsin B; *PI3K*, phosphoinositide-3 kinase; *PSMA*, prostate-specific membrane antigen; *HSP*, heat shock protein 47; *Mettl3*, methyltransferase-like 3; *GLUT4*, glucose transporter 4; *VCAM-1*, vascular cell adhesion molecule-1; *HIF-2*, hypoxia-inducible factor-2; *PKM2*, pyruvate kinase M2

## The Regulatory Role of Tumour Microenvironment and Its Impact on Bladder Cancer Angiogenesis

### Non-Coding RNAs and Bladder Cancer Angiogenesis:

#### MicroRNAs

The role of miRNAs has been rigorously investigated since 2006 to identify their molecular networks and target genes in BCs. Several reviews have been published throughout the past few years depicting the significance of miRNAs as potential markers for BC screening and prognostication [79–81, 82••, 83, 84, 85•, 86–88, 89•, 90••]. A few studies have investigated the role of miRNAs in BC angiogenesis (Table 2).

**Table 2:** Studies depicting the role of microRNAs in BC angiogenesis

Publication	microRNA	Sample type	Effect on angiogenesis
Xu et al. [91]	miR-27a	BC tissue samples	The angiogenic factor AGGF1 is downregulated in high-grade carcinomas Hypoxia-induced downregulation of AGGF1 is mediated by miR-27a
Yu et al. [92]	miR-34a	BC tissue samples and BC cell lines SCABER, T24, HT-1376, J82, E, and 5637	miR-34a functions as an anti-metastatic miRNA and suppresses angiogenesis in BC by directly targeting CD44
Wang et al. [93]	miR-124	BC and normal bladder tissue samples BC cell lines J82 and T24 and a human embryonic kidney cell line (HEK 293) Xenografts	miR-124 suppresses expression of UHRF1 through competitive binding of the same region of its 3′-UTR miR-124 overexpression significantly attenuates cellular proliferation, migration, invasion and vasculogenic mimicry in vitro, and tumour growth in vivo
Wu et al. [94]	miR-200c	BC cell lines 5637 and T24 and normal human bladder epithelial cell line, SV-HUC-1	miR-200c could suppress HIF-1α/VEGF expression in BC cells and inhibit angiogenesis, and these regulations were achieved by targeting Akt2/mTOR
Zhou et al. [95]	miR-128	Normal bladder epithelium cell line SV-HUC-1 and BC cell lines T24, 5637, 3-UM-UC-3, RT4 BC tissue samples	miR-128 is significantly downregulated and VEGF-C upregulated in BC tissues and cells
Wang et al. [96]	miR-122	BC cell lines BIU-87, SW780, T24, RT4, and HT1376 and normal bladder epithelial cell line SV-HUC-1 BC tissue samples	miR-122 downregulates VEGFC expression by direct binding to its 3′-UTR
Cao et al. [97]	miR-124	BC tissue samples and BC cell lines T24, 5637, J82, and UM-UC-3 Hek293 and SV-HUC-1	miR-124 significantly inhibits cell viability, decreased angiogenesis rate, prevents cell proliferation, and diminishes the expression of E2F3, CDK4, Ki-67, and VEGF All these changes are reversed by over-expressing CDK4
Zhang et al. [98]	miR-153	BC tissue samples, normal human bladder epithelial cell line, SV-HUC-1, and BC cell lines UMUC3, 5637, T24, and J82	miR-153 inhibits BC angiogenesis in vivo and in vitro by targeting indoleamine 2,3-dioxygenase 1
Wang et al. [99]	miR-942-3p	BC and normal bladder tissue specimens Cell lines SV-HUC-1, HEK-293 T, and HUVECs and the human BC cell lines 5637, J82, T24, EJ, TCCSUP, RT4, and UM-UC-3	TAZ induces upregulation of miR-942-3p expression by inhibiting the expression of large tumour suppressor 2. MiR-942-3p attenuated the impacts on cell proliferation, angiogenesis, EMT, glycolysis, and ROS levels induced by TAZ knockdown
Chan et al. [100]	miR-429	Clinical tissue samples and human cell lines, BFTC909 and TCCSUP Phoenix-AMPHO cell line	Higher level of VEGFA or MVD has a positive correlation with the expression of CEBPD and a negative relation to hsa-miR-429 and leads to tumour aggressiveness with worse disease-specific, metastasis-free survival

*AGGF1*, angiogenic factor with G-patch and forkhead-associated domain 1; *UGFR*, ubiquitin-like with PHD and RING finger domain 1; *mTOR*, mammalian target of rapamycin; *CDK4*, cyclin-dependent kinase 4; *TAZ*, transcriptional coactivator with PDZ-binding motif; *EMT*, epithelial-mesenchymal transition; *ROS*, reactive oxygen species; *CEBPD*, CCAAT/enhancer-binding protein delta

#### Long non-coding RNAs

Long non-coding RNAs (lncRNAs) are non-protein coding RNAs with more than 200 nucleotides in length with the ability to down/upregulate gene expression [101–103]. There are more than 100 dysregulated lncRNAs in BC [104••]. LncRNAs exhibit tumour-suppressor and tumour-promoting roles, tightly regulating apoptosis, glycolysis, and EMT in BC. LncRNAs regulate immune cell infiltration in the tumour microenvironment and affect the response of BC cells to immunotherapy. lncRNAs are also able to regulate microRNAs, STAT3, Wnt, PTEN, and PI3K/Akt pathways affecting both the proliferation and migration of BC cells [105••, 106••]. Several studies have investigated the role of lncRNAs in BC angiogenesis (Table 3).

**Table 3:** Studies depicting the role of lncRNAs in BC angiogenesis

Publication	lncRNA	Sample type	Effect on angiogenesis
Chi et al. [107]	RP11-79H23.3	BC tissue samples and normal bladder epithelium cell line SV-HUC-1 and BC cell lines (EJ, T24, BIU87)	RP11-79H23.3 suppresses tumourigenesis, metastasis, and angiogenesis of BC cells in vivo Downregulation of RP11-79H23.3 leads to higher CD31 and S100A4 expression and more microvessels. RP11-79H23.3 can regulate the expression of the miR-107/PTEN axis and activate the PI3K/AKT signalling pathway to contribute to the proliferation, migration, apoptosis, and angiogenesis of BC cells
Liu et al. [108]	FAM83H-AS1	BC cell lines T24, SW780, HT-1197, BK10, and HTB-9 and normal urothelial cell line SV-HUC-1 BC tissue samples	FAM83H-AS1 promotes growth, metastasis, and angiogenesis of BC cells through c-Myc-mediated ULK3 upregulation and the Hedgehog pathway activation
Wang et al. [109]	LINC00482	BC tissue samples and BC cell lines HT-1376, T24, 5637(HTB-9), and J82 as well as human normal bladder epithelial cells (SV-HUC-1)	LINC00482 induces the expression of MMP15 by interacting with FOXA1, thereby contributing to the inflammation and angiogenesis in BC
Li et al. [110•]	KIRREL1IT1, AC005625.1, AC018809.1, AC008760.1 and AC083862.2	BC tissue samples and SV-HUC-1, BC cell lines (T24, UM-UC-3, 5637, J82, and TCC-SUP) and HUVEC	Five angiogenesis-related long non-coding RNAs are identified and a reliable and accurate angiogenesis-related risk score model is provided
Xiao et al. [111]	LINC00958	BC cell linesT24 and J82 cells and human normal urothelial SV-HUC-1 as well as BALB/c-nu nude mice and clinical blood samples from BC patients	LINC00958 inhibits autophagy of BC cells via sponge adsorption of miR-625-5p to promote angiogenesis and oxidative stress
He et al. [112]	TPRG1-AS1	Human BC cell lines: RT4, T24, J82 and 5637 SV-HUC-1 and Xenografts	TFAP2A promotes the transcription of TPRG1-AS1. TPRG1-AS1 reversed the inhibitory effect of TFAP2A knockdown on glycolysis and angiogenesis in BC cells. TPRG1-AS1 inhibits the transcription of CRTAC1 by recruiting a DNA methyltransferase to the promoter of CRTAC1 and increasing the DNA methylation of its promoter. CRTAC1 inhibits glycolysis and angiogenesis in BC cells
Kang et al. [113]	lncRNAs (USP30-AS1, LINC02321, PSMB8-AS1, KRT7-AS, LINC01767, and OCIAD1-AS1)	Bioinformatics analysis BC RNA-sequencing and clinical data were downloaded from the TCGA database (https://portal.gdc.cancer.gov/. TCGA-BLCA) lncRNA expression data in BC from the TCGA database were assessed using scripts written using the “Perl” (version 5.32.1.1) programming language	Six angiogenesis-associated lncRNA signatures reported in the study may be used to predict the prognosis of patients with BC, and LINC02321 promoted malignant progression of BC via the VEGFA signalling pathway

*PTEN*, phosphatase and tensin homolog; *FOXA1*, forkhead box A1; *TFAP2A*, transcription factor AP-2 alpha

#### Circular RNAs

Hundreds of circular RNAs (circRNAs) are significantly dysregulated in human BC tissues [114]. Most circRNAs regulate BC through miRNA sponging regulatory mechanisms. Most have been reported to be associated with many clinicopathologic characteristics of BC, including tumour size, grade, differentiation, and stage; lymph node metastasis; tumour numbers; distant metastasis; invasion; and recurrence [115••]. Studies investigating the role of circRNAs in BC angiogenesis are presented in (Table 4).

**Table 4:** Studies depicting the role of circRNAs in BC angiogenesis

Publication	circRNA	Sample type	Effect on angiogenesis
Li et al. [114]	circHIPK3	Clinical tissue samples and BC cell lines, T24T, T24, UMUC3 and human immortalised uroepithelium cell line SV‐HUC‐1 and endothelial cell line HUVEC	Overexpression of circHIPK3 effectively inhibits migration, invasion, and angiogenesis of BC cells in vitro*.* circHIPK3 can abundantly sponge miR-558 to suppress the expression of heparanase
Zhong et al. [116]	circRNA-MYLK	Clinical tissue samples, BC cell lines EJ, T24, 5673, and BIU-87 and xenograft mouse model	circRNA-MYLK could directly bind to miR-29a and relieve suppression for target VEGFA, which activated VEGFA/VEGFR2 signalling pathway
Cao et al. [117]	circ0001429	Clinical tissue samples Cell culture: T2, 5637, BIU-87, SV-HUC-1 Xenograft mouse model	Circ0001429 regulates progression of BC through binding miR-205-5p and promoting VEGFA expression
Mao et al. [118]	Hsa_circ_0068871	Clinical tissue samples, BC cell lines T24, UMUC3, EJ, and J82 and the immortalised human normal bladder epithelial cell line SV-HUC-1 and xenograft mouse model	hsa_circ_0068871 upregulates FGFR3 expression and activates STAT3 by targeting miR-181a-5p to promote BC progression
Wei et al. [119]	Has_circRNA_403658	Normal bladder epithelial cells CCC-HB-2 and BC cell lines: SW780, 5637, T24, J82, and RT4, clinical tissue samples and xenograft mouse model	circ403658 is induced by HIF-1α and increases the expression of VEGFR and EGFR
Wang et al. [120]	circSEMA5A	BC tumour and normal tissues Human BC cell lines T24, UM-UC-3, 5637, J82, the normal human uroepithelial cell line SV-HUC-1 and human umbilical vein endothelial cells HUVEC	circSEMA5A promotes proliferation, suppresses apoptosis, migration, accelerates invasion, enhances angiogenesis, and promotes glycolysis of BC. circSEMA5A serves as a miRNA sponge for miR-330-5p to upregulate Enolase 1 expression and facilitates the activation of Akt and β-catenin signalling pathways

### Metabolic Derangements and Angiogenesis in Bladder Cancer

Recent studies demonstrate a link between abnormal metabolic processes and uncontrolled angiogenesis in BCs. In one study, the role of small extracellular vesicles in reprogramming glucose metabolism by increasing hexosamine biosynthesis pathway flux in endothelial cells in response to glutamine fructose-6-phosphate aminotransferase (GFAT) is demonstrated suggesting that inhibiting small extracellular vesicle-mediated GFAT1 secretion from BC cells may serve as novel anti-angiogenetic therapy [121•]. Hepatitis B X-interacting protein (HBXIP), a marker associated with a poor prognosis for BC, was shown to reduce glycolysis in BC cells via regulation of AKT/mTOR signalling, thereby blocking BC angiogenesis. This study provides a potential strategy to target HBXIP and AKT/mTOR for regulating glycolysis progression concurrently with anti-angiogenesis effects [122].

Cholesterol metabolism plays a significant role during cancer progression. The farnesoid X receptor (FXR) is a bile acid-activated transcription factor and a member of the nuclear receptor superfamily. Two studies have shown that FXR contributes to BC cell migration, invasion, and angiogenesis through the proteasomal degradation pathway. FXR overexpression induces AMPK phosphorylation and decreases cholesterol synthase-related protein expression. Statin usage showed potent enough efficacy to strengthen the enhancement of FXR-inhibited migration, adhesion, and angiogenesis in human urothelial carcinoma cells [123, 124].

### Immunologic Factors: Relation to Angiogenesis in Bladder Cancers

BCs are considered as highly immunogenic malignancies capable of manipulating the host immune system to facilitate the survival and progression of their cancerous cells [125].

In BCs, both innate and adaptive immunity have been shown to contribute to carcinogenesis. TAMs, myeloid-derived suppressor cells, T cells, B cells, and their associated cytokines and chemokines have been shown to play a role in bladder carcinogenesis [126]. Of these, TAMs have been shown to directly contribute to angiogenesis in a few studies.

#### Tumour-Associated Macrophages

Multiple studies have shown that TAMs can produce multiple angiogenic factors including VEGF, TNF-α, IL-1β, IL-8 (CXCL8), PDGF, bFGF, thymidine phosphorylase, and MMPs [127]. Several studies have investigated the relationship between BCs and TAMs including their effect on BCG (Bacillus Calmette Guerin) treatment in in situ carcinomas, prognostic significance, the mechanisms involved in their activation and polarisation and their role in angiogenesis [126]. Hanada et al. assessed TAMs and micro-vessel counts in 63 patients with BC. Immunohistochemistry using anti-CD34 antibody was used to identify micro-vessels, and anti-CD-68 antibody was used to identify TAMs in tumour tissues. The results showed that TAMs and micro-vessel counts values in invasive BCs were significantly higher than in superficial tumours. There was also a positive correlation between TAM count and micro-vessel count in this study suggesting a prognostic role of TAM count in BC [128].

In 2016, Takeuchi et al. investigated TAM and micro-vessel counts in 17 NMIBC and 4 invasive cases. M2 macrophages were identified by immunohistochemical staining with anti-CD-68 and anti-CD163 antibodies identifying both the macrophage lineage and an M2-specific surface receptor, respectively. Micro-vessel counts were determined using immunohistochemistry with anti-CD34. Their results showed that the higher ratio of CD163^+^/CD68^+^ macrophages in the stroma, tumour, and total tumour tissues was significantly correlated with a higher stage and grade. In addition, the low ratio of CD68^+^/CD34^+^ micro-vessels was significantly correlated with a higher stage. There was also a positive correlation between TAMs and micro-vessel counts [129]. Furthermore, a study investigating the role of CXCL8 secreted by TAMs in urothelial carcinoma showed the infiltration of TAMs in the tumour microenvironment led to the elevation of CXCL8, which in turn promoted the secretion of MMP-9, VEGF, and E-cadherin by BC cells. CXCL8 altered the migration, invasion, and pro-angiogenic capacity of BC cells and accelerated cancer progression [130].

#### Chronic Inflammation and Angiogenesis in Bladder Cancer

BC is a chronic inflammation-associated type of neoplasia. To this effect, studies have shown that chronic inflammation whether local or systemic increases the risk of developing BCs [131]. BC cells have been shown to secrete various pro-inflammatory molecules that contribute to their advancement through increasing proliferation, angiogenesis, invasion, and metastasis [132].

Several signalling pathways have been linked to the initiation and progression of BCs during inflammation, including COX-2/nitric oxide synthase (NOS), janus activated kinase (JAK)-STAT3, the nuclear factor-kappaB (NF-*κ*B), and PI3K-Akt-mTOR [131]. Nitric oxide generation from inducible isoform of nitric oxide synthases in the malignant epithelium and from endothelial isoform in tumour stroma has an important potential in the angiogenesis of BC [10]. NF-*κ*B activation also mediates angiogenesis and metastasis in BC through the regulation of IL-8 [133].

Several pro-inflammatory cytokines have been associated with BC pathogenesis, but a few have been linked to angiogenic activity in these tumours. IL-6 was found to promote angiogenesis and vascular modelling via VEGF and STAT3, which affects the genes mediating angiogenesis. IL-6 silencing vector attenuated angiogenesis as demonstrated by the staining of CD31 and VEGF [134]. TNF-α also promotes angiogenesis and the development of several tumour types. In a study by Feng et al., expressional changes of Pigment epithelium-derived factor (PEDF) and TNF-α were related to angiogenesis of bladder tumours. TNF-α expression was positively correlated with MVD, while PEDF was negatively correlated with MVD [135]. Studies have demonstrated that IL-8 expression enhances angiogenic activity through the induction of MMP9 and subsequently regulates the tumourigenesis and production of spontaneous metastases in BCs [136]. The levels of human neutrophil peptide (HNP)-1, -2, and -3, produced by neutrophils, were found to be increased in BC with an effect on tumour angiogenesis and growth. All three HNPs are subtypes of α-defensins, proteins that aid in the recruitment of leukocytes. The indirect effects of HNPs 1–3 include stimulation of tumour cell proliferation and potentially tumour angiogenesis [137].

CD74 and macrophage migration inhibitory factor (MIF) were found to be expressed in MIBC samples, and only one high-grade BC cell line, HT-1376, compared with normal, NMIBC samples. The tumourigenesis and MVD assays indicated less proliferation and angiogenesis in the knockdown-HT-1376 cells [138]. Angiogenin (ANG), a member of the RNase A superfamily, has been demonstrated to promote tumour angiogenesis and metastasis in BC by activating key downstream target molecules of the PI3K-AKT-mTOR signalling pathway [139]. The urinary levels of ANG and angiostatin and the marker of oxidative stress, 8-iso-prostaglandin F2*α* (8-iso-PGF2*α*), the tumour progression marker *ɣ*-synuclein as well as IL-13 were shown to increase with the development of BC. These results further strengthen the interactive relationship between angiogenesis, oxidative stress, and inflammation in the pathogenesis and development of BC [140, 141]

## Vascular Mimicry as a Distinct Angiogenic Mechanism in Bladder Cancers

VM was originally described in melanomas [142]. The term denotes the formation of microcirculatory systems within tumour tissues that do not require the presence of endothelial cells. VM vessels are instead formed by tumour cells creating channels that connect directly with the normal surrounding blood vessels to deliver erythrocytes and nutrients to tumourous tissues [143]. The cellular and molecular events underlying the formation of VM remain unclear despite many studies investigating factors related to cell migration, invasion, and matrix remodelling and their relation to VM formation [144].

### In vivo* Characterisation of Vascular Mimicry*

In vivo characterisation of VM vessels was initially assessed by histological examination of tumour tissues/xenografts stained with CD34/CD31 combined with PAS staining where VM channels appear as tubule-like structures containing red blood cells. The tubules are positive for PAS staining and negative for CD34/CD31 contrasting with surrounding endothelium-dependant vessels which are positive for routine endothelial cell markers [142, 145]. These channels have also been shown to express elevated levels of genes associated with the multipotent, stem cell-like phenotype [146–148].

Based on PAS staining, VM vessels can present as straight channels, parallel straight channels, parallel straight channels with cross links, open arcs, arcs with branching, closed loops and networks. The vessels are matrix-rich structures, rich in laminin, proteoglycans, heparan sulphate, and collagens IV and VI as a part of their basement membranes visualised by the PAS staining. The tubular formations are sometimes referred to as “pattern structures” [149, 150].

Further studies into VM revealed more markers differentiating VM cells from normal endothelial cells including TIE-2, TIE-1, VEGFR-1 and 2, P-selectin, VCAM, CD106, Neuopilin, endoglin, and LAMC2, to name a few [151••]. Although the presence of PAS + tubular structures containing RBCs is accepted as an indicator of VM in the literature, it is believed that it cannot be independently used as a definitive proof of VM [152]. PAS + structures have been suggested to cross over true blood vessels containing RBCs, and cancer cells can secrete copious amounts of PAS + mucoproteins, but this should not necessarily imply vessel formation. Electron microscopy studies have not shown blood components inside VM channels in some studies, and many studies have failed to provide adequate imaging evidence of VM [153, 154]. These findings have raised scepticism about the methods used to identify VM in many studies in the literature [152].

There is currently no definitive VM marker that characterises non-endothelial vessels. More studies are needed to explore potential panels of biomarkers to improve the identification of VM vessels in vivo and in vitro.

### In Vitro* Characterisation of Vascular Mimicry*

In vitro characterisation of VM was initially assessed on models using a 3D matrix (Matrigel) where the presence of intercellular connections was used as evidence for VM [155]. The concept was then reinforced by Francescone et al. characterising a Matrigel-based tube formation assay to assess VM in tumours. Ever since, this article has been cited as a reference for validating the use of intercellular connections as evidence of VM [156]. Notably, most of the studies investigating VM in vitro have utilised intercellular connections formed between cancer cells to report the presence and mechanisms of this phenomenon [152].

Valdivia et al. outline a strong argument that intercellular connections in an in vitro model do not necessarily represent fluid-containing vessels. In their article, the authors provide ample evidence based on a thorough literature review suggesting that most structures presented as VM in the literature may not in fact contain a lumen and thus cannot be regarded as fluid conducting vessels. The authors further highlight that only a few in vitro studies have persuasively demonstrated a functional lumen in tubular structures [157–164]. Building on these studies, the authors describe an in vitro model that utilises Matrigel to demonstrate tubular structures with microinjected trypan blue dye to illicit the movement of fluid. The use of confocal microscopy and IMARIS (Microscopy Image Analysis Software) reconstruction further confirms the presence of a lumen and a glycoprotein-rich layer flanked by cancer cells in their suggested model [152].

### Vascular Mimicry in Bladder Cancer

The presence of VM in BCs has been investigated in a few studies. In these studies, VM was either assessed in vivo and/or in vitro using the classical methods described above.

In a study assessing the impact of VM on recurrence-free survival in urothelial carcinoma of the bladder, it was concluded that VM seemed to predict the risk of developing lung metastases after radical cystectomy. The combination of VM and TNM stage showed a better prognostic value than TNM stage alone or VM alone. The presence of VM also identified a subgroup of patients with MIBC who appeared to benefit from adjuvant chemotherapy. VM vessels in this study were identified using CD31-PAS double staining particularly if they contained RBCs [165].

In an earlier study, ECV304 human BC cells were used to determine how tumour cells take part in tumour neovascularisation. Subcutaneous ECV304 xenografts in mice showed various vessel types, including angiogenic vessels, tumour cell-related vessels, and extracellular matrix networks. ECV304 cells, cultured on collagen I gels, formed tube networks with the expression of several endothelial-related markers. The study concluded that ECV304 cells possess characteristics which confer the ability to mimic endothelial cells and facilitate the formation of VM. Vessels in this study were characterised using CD34, CD31, vWF, and Azan staining [166].

In a subsequent study, the cell line currently known as T24/83 was used to create a model of in vitro vasculogenic mimicry. In co-cultures of ECV304 and C378 human fibroblasts, tubular structures were identifiable after 8 days. The tubular structures showed elevated levels of transglutaminase 2 (TG2) antigen and TG2 in situ activity. In situ activity for TG2 showed co-localisation with both fibronectin and collagen IV. Deposition of these proteins into the extracellular matrix was reduced by the inclusion of non-cell penetrating TG inhibitors. Incubation of ECV304 cells with these same irreversible inhibitors reduced cell migration which paralleled a loss in focal adhesion assembly, actin cytoskeleton formation, and fibronectin deposition. The study concluded that TG2 appears to be essential for ECV304 tube formation, thus representing a potential novel therapeutic target in the inhibition of VM [167].

Yu et al. explored the expressions of CD133 and CD82/KAI1 in urothelial carcinoma and their relation to VM. Using immunohistochemistry, the positivity rates for these markers were significantly different between normal bladder epithelium and urothelial carcinomas where CD82 was downregulated in carcinomas and CD133 showed upregulation in cancerous tissues. Positive expressions of CD133, CD82/KAI1, and VM were significantly correlated with pTNM stage and tumour relapse but not with gender, age, or tumour numbers. Respectively, CD133 expression was positively correlated with VM, and CD82/KAI1 expression was negatively correlated with VM and CD133 [168].

In a study using BC cell lines UM-UC-3 and J82, and the immortalised human bladder epithelium cell line SV-HUC-1; 3-D cultures were constructed to detect VM formation. UM-UC-3 and J82 cells exhibited VM formation; however, SV-HUC-1 did not. Furthermore, VM-forming cancer cell lines UM-UC-3 and J82 exhibited higher zinc finger E-box binding homeobox 1 (ZEB1) expression. VM was observed in 31.1% of specimens from BC tissues, and cases with high ZEB1 expression accounted for 60% of patients. In addition, ZEB1 expression was significantly associated with VM and increased as the grade and stage of the tumour developed. The study concluded that ZEB1 may be associated with VM in BC and serve a key role in the process of VM formation [145].

Finally, androgen receptor was found to increase BC metastasis through activating VM formation. This was evidenced through altering the expression of the VM marker SLPI (secretory leukocyte protease inhibitor) through miR-525-5p which was decreased via binding to different androgen-response-elements located at distinct positions in the miR-525 precursor promoter [169].

The potential of VM as a therapeutic target in advanced high-grade BCs and in anti-angiogenic refractory patients remains to be investigated. The field of VM is currently marred with controversy demanding the development and standardisation of assays for the detection and quantification of VM in both in vitro and in vivo conditions. Additional studies are also needed to further characterise biomarkers/pathways of VM in BCs.

## Perspectives and Concluding Notes

The studies summarised in this review undoubtedly illustrate the exponential evolution of current understanding of the tumour ecosystem. While VEGF, FGF, and PGF remain as the quintessential angiogenic molecules, the volume of suggested angiogenic regulators depicts a picture of increasing complexity and necessitates an integrative approach targeting angiogenesis at the structural, functional, and molecular levels. The tumour microenvironment holds the key for the future advancement of cancer detection and prognosis. While TAMs play a significant role in BC angiogenesis, the potential of reprogramming TAMs to induce vessel normalisation in BCs is yet to be fully investigated [170••].

There is a need for reliable and non-invasive biomarkers for assessing angiogenic activity in BCs. Current evidence strongly points to the role of non-coding RNAs in BC aetiopathogenesis. The abundance, conservation, and stability of non-coding RNAs render them potential effective diagnostic and prognostic biomarkers for BC. Analytical difficulties exist due to low expression levels of many non-coding RNAs and the bias introduced by RNA-seq library preparations [171•]. Exosomes are natural delivery vehicles for angiogenic and anti-angiogenic factors including non-coding RNAs. There is limited understanding of the biologic complexity of exosomes, and further proteomic analyses are needed to characterise their role in the tumour microenvironment. Notably, the shortage of standardised effective methods for exosome isolation, identification, and precise characterisation limits their application in clinical settings [172•].

Recently, extensive development of computational and in vitro experimental models to recapitulate tumour-endothelial cell interactions has posed the potential of a better understanding of the angiogenic process. Microfluidic chips have shown superior potentials for reflecting in vivo geometrical complexities, hydrodynamic stress, and mass transport [173, 174]. These models offer the possibility of creating microenvironments with regional heterogeneity and controllable and quantifiable spatiotemporal gradients [174, 175].

Computational models have accompanied experimental assays from the early days of research on tumour angiogenesis. In silico models of the tumour microenvironment have covered various aspects of tumour-stroma interactions. In contrast to microfluidic chips, in silico models embrace the complexity of a real tumour microenvironment avoiding the shortcomings of in vitro assays [174]. The integrated use of microfluidic models to validate mathematical models promises further opportunities to overcome the limitations inherent to both models. However, the scarcity of experimental and clinical data needed for the building of predictive experimental-theoretical platforms poses a challenge [174].

Ultimately, angiogenesis is a key biological event in BC carcinogenesis. Integration of angiogenic parameters within risk stratification tools will undoubtedly improve prognostication in BCs.
